# Characteristics and Clinical Outcomes of Individuals at High Risk for Pancreatic Cancer: A Descriptive Analysis from a Comprehensive Cancer Center

**DOI:** 10.3390/gidisord1010008

**Published:** 2018-11-01

**Authors:** Griffin P. J. McNamara, Karla N. Ali, Shraddha Vyas, Tri Huynh, Monica Nyland, Deanna Almanza, Christine Laronga, Jason Klapman, Jennifer B. Permuth

**Affiliations:** 1 Department of Cancer Epidemiology, H. Lee Moffitt Cancer Center and Research Institute, Tampa, FL 33612, USA;; 2 Department of Breast Oncology, Moffitt Cancer Center, Tampa, FL 33612, USA;; 3 Department of Gastrointestinal Oncology, Moffitt Cancer Center and Research Institute, Tampa, FL 33612, USA;

**Keywords:** pancreatic cancer, intraductal papillary mucinous neoplasm, mucinous cystic neoplasm, pancreatic intraepithelial neoplasm, mutation carriers, familial pancreatic cancer

## Abstract

Pancreatic cancer (PC), a leading cause of cancer-related deaths in the United States, is typically diagnosed at an advanced stage. To improve survival, there is an unmet need to detect pre-malignant lesions and early invasive disease. Prime populations to study for early detection efforts include cohorts of high risk individuals (HRI): those with increased risk to develop pre-malignant pancreatic cysts and PC because of a familial or hereditary predisposition to the disease and those in the general population of sporadic cases who are incidentally found to harbor a pre-malignant pancreatic cyst. The objective of this study was to describe the characteristics and clinical outcomes of cohorts of HRI identified at Moffitt Cancer Center. We set out to determine the uptake of screening, the prevalence and characteristics of solid and cystic pancreatic lesions detected via screening or as incidental findings, and the age at which lesions were detected. Of a total of 329 HRI, roughly one-third were found to have pancreatic lesions, most of which constituted pre-malignant cysts known as intraductal papillary mucinous neoplasms. Individuals with the highest genetic risk for PC were found to have smaller cysts at a much earlier age than sporadic cases with incidental findings; however, many individuals at high genetic risk did not have abdominal imaging reports on file. We also identified a subset of HRI at moderate genetic risk for PC that were found to have cystic and solid pancreatic lesions as part of a diagnostic work-up rather than a screening protocol. These findings suggest the pancreatic research community should consider expanding criteria for who should be offered screening. We also emphasize the importance of continuity of care between cancer genetics and gastrointestinal oncology clinics so that HRI are made aware of the opportunities related to genetic counseling, genetic testing, and screening.

## 1. Introduction

Pancreatic ductal adenocarcinoma (PDAC), commonly known as PC, is currently the third leading cause of cancer-related deaths in the United States and has a 5-year relative survival rate of 8–9%, the lowest of any malignancy [1–4]. In 2018, approximately 55,440 new cases of PC will be diagnosed and 44,330 patients will die from PC [1]. Unfortunately, 80–85% of patients are diagnosed with late stage, inoperable disease because symptoms did not appear until the disease had metastasized [5]. Symptoms include jaundice, abdominal pain, and weight loss, whereas earlier stages are typically asymptomatic [6]. To improve PC survival, there is a dire need to develop screening approaches to detect early stage, operable malignancies. Given that the lifetime risk for developing PC is only 1.5% [7], screening the general population is impractical; however, selectively screening individuals at increased risk for PC can enable detection of early-stage malignancies and even pre-malignant lesions and prolong survival [8].

### 1.1. Risk Factors for PC

It is estimated that approximately 25% of PDAC cases can be attributed to environmental and lifestyle risk factors [9]. In addition to age, the most established risk factors for PC are tobacco exposure, heavy alcohol use (>60 mL ethanol/day), and a personal history of obesity, pancreatitis, and/or diabetes [9,10]. Approximately 10% of PC cases develop because of a familial or hereditary predisposition, which place them at a 1.8- to 132-fold higher risk than individuals in the general population [11–13]. Thus, genetically high-risk individuals (HRI) are a prime population for early detection efforts [11,12,14–18]. HRI can be defined as having “familial PC” when they have two or more first degree relatives (FDR) with the disease or have one FDR and at least two affected second degree relatives (SDR) [7]. The lifetime risk for developing PC increases with the number of FDR with the disease, and spans from 3% with one affected FDR, 8–12% with two affected FDR, and up to a 40% lifetime risk with three affected FDR [7,12]. As summarized in Table 1, deleterious mutations in genes associated with a hereditary cancer syndrome or chronic inflammation of the pancreas also pose significant risks for PC development [16,19]. Of PDACs associated with hereditary PC syndromes, most (around 5–19%) are attributed to mutations in *BRCA2* [17] which confer a 3.5–10-fold increase in risk [20–22].

### 1.2. Pre-Malignant PC Precursors Exist and Can Be Detected via Imaging

It is now established that PC can develop from three main precursor lesions: pancreatic intraepithelial neoplasia (PanIN), intraductal papillary mucinous neoplasms (IPMN), and mucinous cystic neoplasms (MCN) [32]. While PanINs are microscopic and are typically only viewed pathologically, IPMNs and MCNs are macroscopic lesions that can be detected via radiologic imaging with endoscopic ultrasound (EUS), magnetic resonance imaging (MRI), or computed tomography (CT) [33–38]. Most IPMNs and MCNs are detected incidentally among individuals in the general population who undergo cross-sectional imaging for reasons unrelated to their pancreas and are often thought to be ‘sporadic’ or non-familial. However, PC precursors are also commonly detected in familial kindreds and as part of hereditary cancer syndromes (Table 1) [2,14,19,23–26,28–31,34,39]. For example, in a small Israeli study involving 51 patients with IPMNs who underwent genetic testing, 25% of cases with a family history of PC were found to have *BRCA2* mutations [23]. In a study of 79 *P16*/*CDKN2A* carriers who underwent MRIs in the Netherlands, 11% (*n* = 7) primarily had side branch duct IPMNs detected [25]. Most IPMNs with side branch duct involvement have a lower risk of malignant transformation than main pancreatic duct IPMNs, with mean frequencies of high-grade disease or invasive carcinoma of 31% and 62%, respectively [40]. Those with main pancreatic duct involvement are typically recommended for surgical removal because of their significant malignant potential, whereas side-branch duct IPMNs are typically observed unless they occur with concerning radiologic features [14,41]

Based on recommendations from the International Cancer of the Pancreas Screening (CAPS) consortium, HRI with a 5–10% lifetime risk for developing PC should be offered screening for pancreatic masses as part of a research study using EUS, MRI, or both. CT is not opportune for screening unaffected individuals because of exposure to radiation [7,42]. Current CAPS guidelines recommend screening those with at least two affected FDRs; patients with Peutz-Jeghers syndrome (PJS); and *P16*, *BRCA2* and hereditary non-polyposis colorectal cancer (HNPCC) mutation carriers with ≥1 affected FDR [7]. The proper age for screening, however, remains a topic of debate. Although experts from CAPS do not make specific recommendations regarding the proper age to initiate screening, some studies have recommended screening at 55 years of age or 10 years younger than the closest relative with PDAC [7,43], while others have shown diagnostic yield is highest in individuals >65 years [7,44]. It is noteworthy that HRI with PJS are prone to developing PDAC at a much younger age and require earlier screening [16].

The objective of the current study was to describe characteristics and clinical outcomes of cohorts of HRI identified at H. Lee Moffitt Cancer Center and Research Institute (Moffitt), the only National Cancer Institute-designated Comprehensive Cancer Center based in the state of Florida. Specifically, we set out to determine the uptake of screening, the prevalence and characteristics of solid and cystic lesions detected via screening or as incidental findings, and the age at which lesions are detected.

## 2. Results

### 2.1. Characteristics of the Study Cohort

We identified 329 unique individuals at risk for PC who were seen at Moffitt and consented to one of three IRB-approved protocols leveraged for this analysis (see Methods). Individuals were classified into three groups of HRI: (1) Those meeting the 5–10% lifetime risk of PC mentioned in CAPS guidelines (*n* = 105): including 45 mutation carriers who reported a FDR with PC and 60 individuals with familial PC and no known mutation; (2) those not meeting the CAPS guidelines threshold (*n* = 158): including 11 individuals who have one affected FDR and 147 individuals with a known deleterious mutation and no family history of PC; and (3) individuals with incidental findings of pancreatic lesions that had no known family history of PC or a hereditary syndrome (“sporadic cases”, *n* = 66) (Supplementary Figure S1). Characteristics of the entire study population, stratified by the HRI group, are included in Table 2. Nearly three-quarters of the study population were female (*n* = 243, 73.9%), primarily due to a much higher number of women seeking genetic counseling and testing for *BRCA1*/*2* mutations. The majority of our population was Non-Hispanic, White (*n* = 277, 84.2%). Almost 59% of individuals had never smoked (*n* = 192) and 18.5% (*n* = 61) had a personal history of cancer, pancreatitis, or diabetes.

Almost half of the individuals (*n* = 157, 47.7%) did not have evidence of abdominal imaging in their electronic medical record. Of 172 individuals that had abdominal imaging reports on file, 107 (62%) had cystic or solid pancreatic lesions detected, most of which were IPMNs. The mean age at lesion diagnosis was significantly different across the three groups (*p*-value = 0.0165); on average, sporadic cases were older (67.6 years) than individuals meeting CAPS criteria (62.6 years) and those at elevated risk who did not meet CAPS criteria (60.7 years) (Table 2). Lesion size was significantly different between HRI groups (*p* < 0.0001), with the greatest lesion size observed for sporadic cases (2.62 cm) followed by individuals not meeting CAPS guidelines (1.95 cm) and those meeting CAPS guidelines (0.72 cm) (Table 2). Not surprisingly, because of their small size, approximately 40% of the lesions identified during surveillance of HRI meeting CAPS guidelines were an unclassified type.

### 2.2. Outcomes of HRI that met CAPS Guidelines

**Figure 1.**
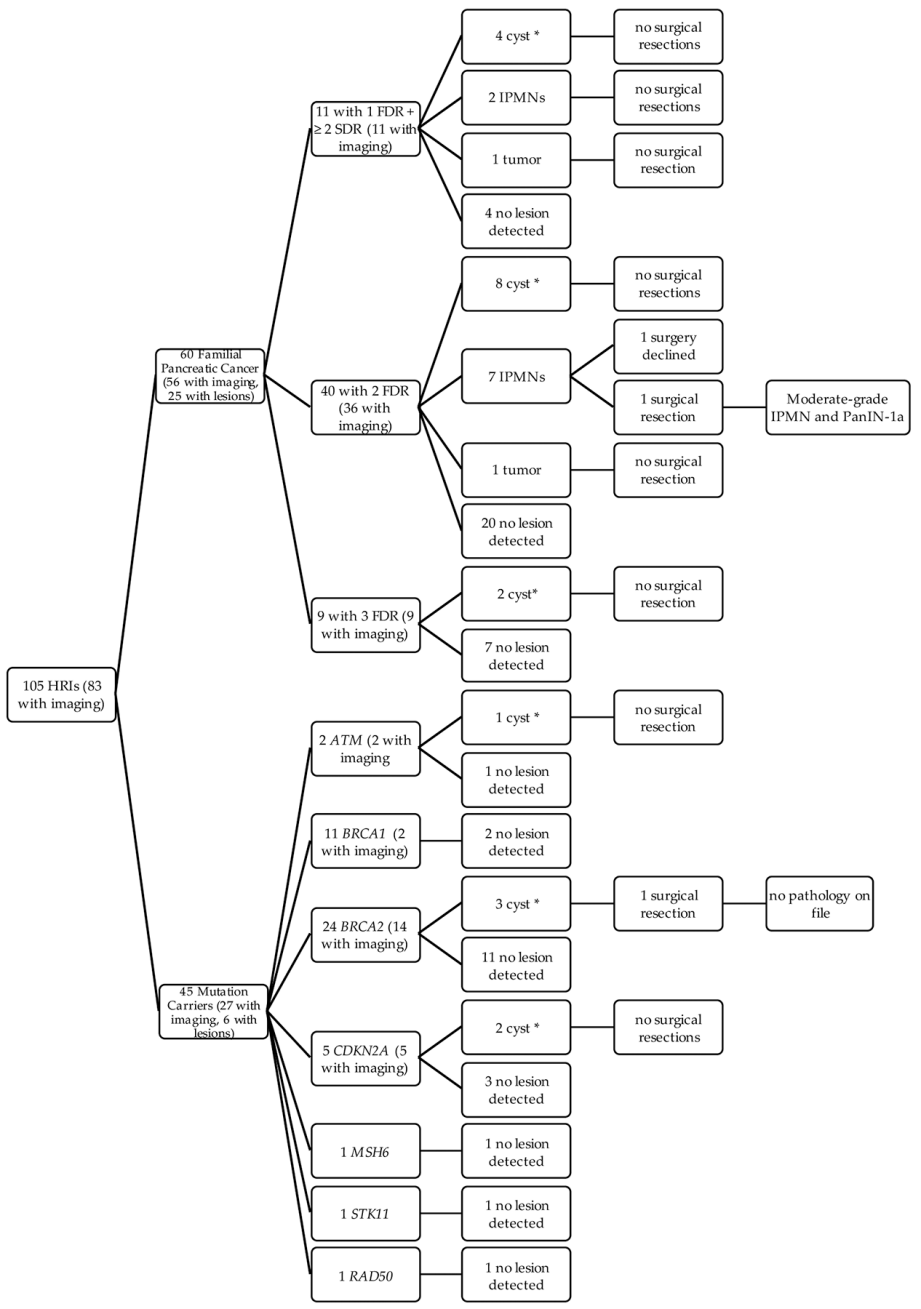
Breakdown of high risk individuals meeting CAPs guidelines and the yield of pancreatic lesions detected. * cyst type not specified.

Of the 105 HRI that met CAPS guidelines, most (*n* = 83, 79.1%) underwent imaging of the pancreas at Moffitt or had outside imaging on file. Thirty-one of these 83 individuals with imaging (37.4%) were found to have pancreatic lesions (Figure 1). Of the 60 familial cases, nearly all (*n* = 56 or 93.3%) had abdominal imaging reports on file. Twenty-five of the 56 familial cases (44.6%) had lesions detected, including two cancerous tumors and 9 IPMNs, one of which was resected and found to harbor moderate-grade disease and PANIN-1A (Figure 1). On the other hand, only 27 (60.0%) of the 45 mutation carriers meeting CAPS criteria had abdominal imaging on file. Six of the 27 mutation carriers (22%) had pancreatic cysts of unknown type detected, including 1 *ATM* carrier, 3 *BRCA2* carriers, and 2 *CDKN2A* carriers. To our knowledge, all but one of the 105 HRI in this category are still living; the one deceased participant did not have a cystic or solid pancreatic lesion.

### 2.3. Outcomes of HRI That Did Not Meet CAPS Guidelines

**Figure 2.**
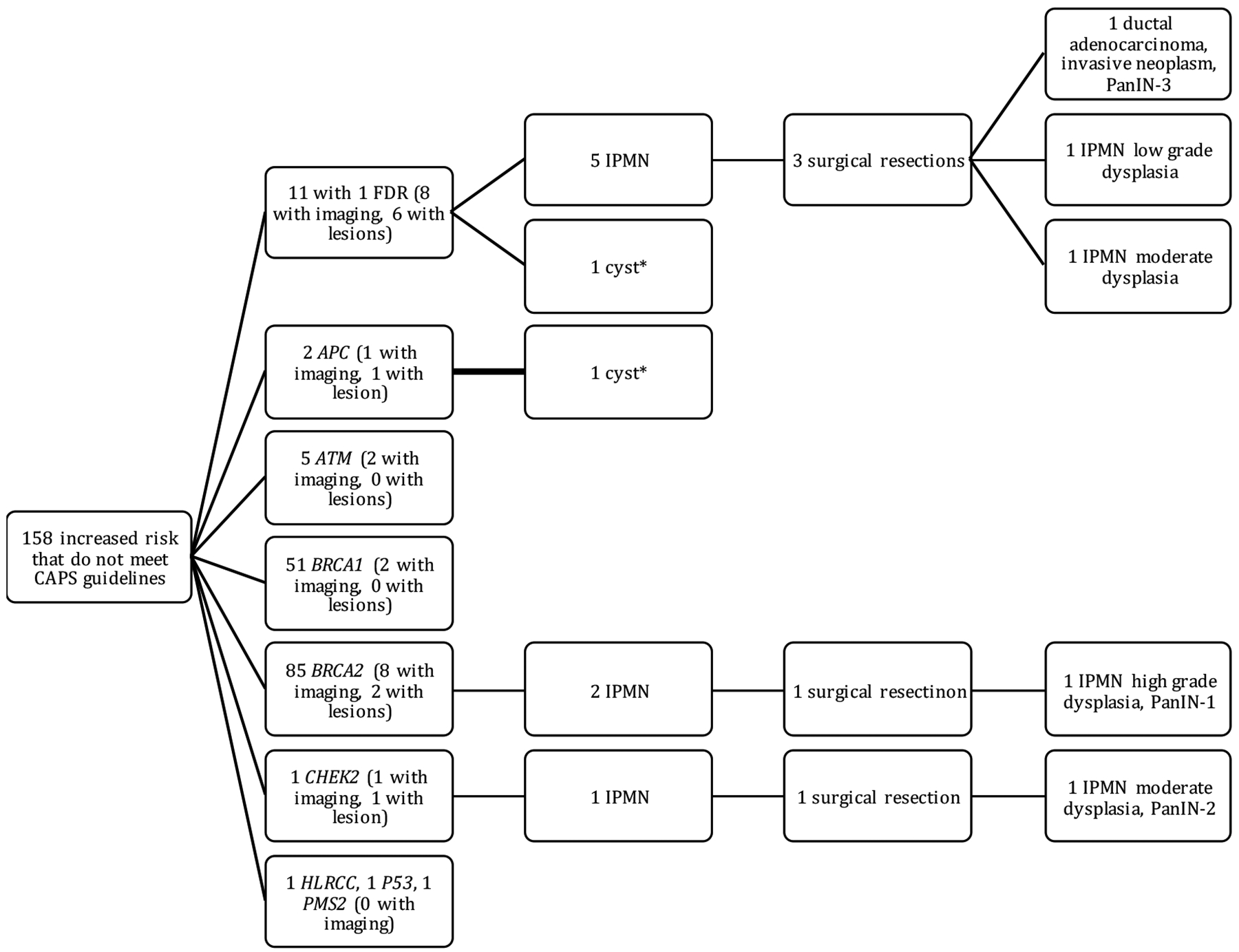
Breakdown of high risk individuals that did not meet CAPS guidelines and the yield of pancreatic lesions detected. These individuals were at increased risk for pancreatic cancer because they had only one first degree relative with pancreatic cancer or a known deleterious mutation (with no family history of pancreatic cancer). * cyst type not specified.

Of the 158 patients who were at increased risk for PC but did not meet CAPS guidelines for screening (Figure 2), 11 had one affected FDR and 147 were known mutation carriers with no known family history of PC. Of the 11 individuals with one affected FDR, eight had imaging on file. Six of these eight individuals had lesions, five of which were IPMNs. Three of these individuals underwent surgical resection, resulting in one adenocarcinoma with PanIN-3, one IPMN with low-grade dysplasia, and one IPMN with moderate-grade dysplasia. Of the 147 mutation carriers, only 23 (15.6%) had abdominal imaging on file and five of these patients were found to carry a 6174 delT *BRCA2* mutation which provides a 10% lifetime risk for PC, nearly double the lifetime risk required for CAPS screening [7,45]. Four of the mutation carriers with imaging on file had lesions; three had IPMNs and one had a cyst of unknown type. Two of the IPMN patients (a *CHEK2* and a *BRCA2* mutation carrier) underwent surgical resection. The IPMN found in the *BRCA2* carrier had main duct involvement seen on imaging. Pathology reports after surgical resection reported that the patient with a *CHEK2* mutation had an IPMN with moderate grade dysplasia, and the patient with a *BRCA2* mutation had an IPMN with high-grade dysplasia and PanIN-1. These lesions were not detected as part of Moffitt’s high-risk surveillance protocol because the participants did not meet CAPS guidelines. Instead, they were detected incidentally among participants recruited as part of our team’s Florida Pancreas Collaborative (FPC) Study (see Methods).

### 2.4. Outcomes of Sporadic Cases

**Figure 3.**
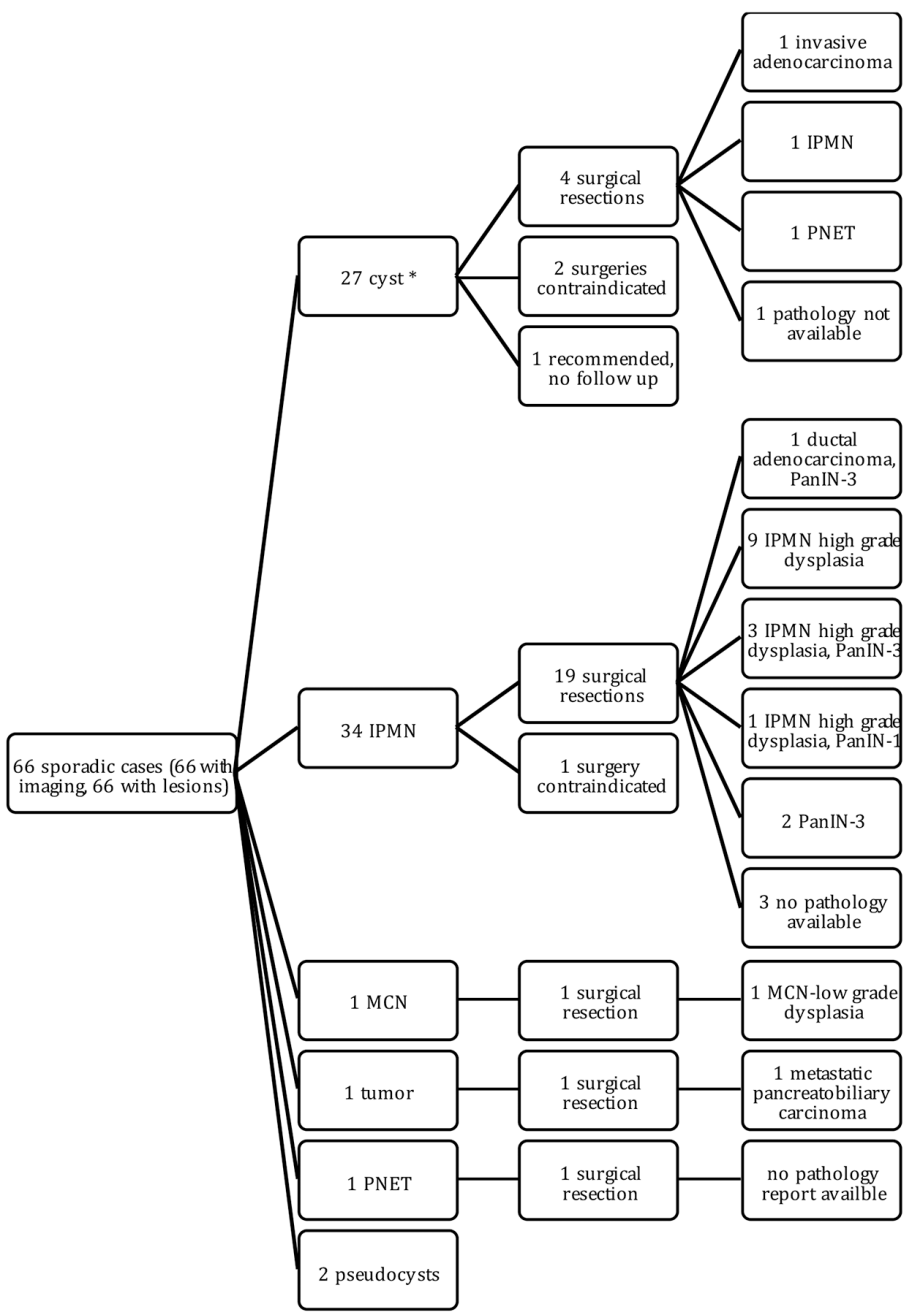
Sporadic cases and pancreatic lesion findings. Individuals in this cohort were identified through the FPC study and have no family history for pancreatic cancer or known deleterious mutation for a hereditary cancer syndrome. * cyst type not specified.

Sixty-six of the 329 HRI in our cohort (20.0%) were considered to be “sporadic” cases, meaning they had no known family history of PC or hereditary syndrome (Figure 3). Most of these cases were recruited through our Florida Pancreas Collaborative Protocol; approximately 10.5% presented with asymptomatic, incidental pancreatic lesions on imaging and the remainder presented with symptoms such as abdominal pain and jaundice. Most of these cases had IPMNs (*n* = 34,51.5%), and 19 of the IPMNs were surgically resected. This yielded one ductal adenocarcinoma with PanIN-3,13 high-grade IPMNs (three of which had an associated PanIN-3 and nine of which had main duct involvement), and two PanIN-3s. Six of the 66 sporadic cases are deceased; three had an IPMN, one with high-grade dysplasia, and three had cysts of unknown types.

## 3. Discussion

Advancements in the early detection and prevention of pancreatic cancer requires strategies to detect and treat pre-malignant lesions and early invasive disease among individuals at high risk to develop this malignancy. We performed a descriptive analysis of the characteristics and clinical outcomes of three groups of high risk individuals (HRI) identified at our cancer center in order to better understand the uptake of screening, the prevalence and characteristics of solid and cystic pancreatic lesions detected via screening or as incidental findings, and the age at which lesions were detected.

As expected, individuals meeting the 5–10% lifetime risk of PC recommended by CAPS had the highest uptake of screening, primarily with a previously-described EUS-only High-risk Surveillance protocol [6]. Nearly 40% of the patients meeting CAPS criteria with abdominal imaging on file were found to have pancreatic lesions, primarily unclassifiable cysts due to their small size, which is consistent with findings from other studies [46]. This suggests that proactive screening efforts for high risk individuals are effective in identifying a high prevalence of smaller pancreatic lesions that should be monitored. Of the 22 individuals for whom we do not have a report of abdominal imaging on file, 18 were at least 50 years old and therefore should be recommended for a screening protocol. Moreover, 11 of these individuals without a report of pancreas screening on file harbored a deleterious *BRCA1* or *BRCA2* mutation and a family history of pancreatic cancer, including one individual with six second degree relatives with the disease. It is possible that these HRI received imaging elsewhere, but our observation highlights the importance of continuity of care between cancer genetics and gastrointestinal oncology clinics so that HRI are made aware of opportunities for screening studies.

Among those at increased moderate genetic risk for PC who did not meet the CAPS guidelines threshold for screening and therefore were not part of a surveillance study at our institution, we identified 10 individuals who had abdominal imaging on file and for whom cystic and solid pancreatic lesions were detected as part of a diagnostic work-up. This is likely an underestimate since most of these patients did not have abdominal imaging on file. We observed an earlier mean age of lesion diagnosis (60.7 years) in this group of HRI compared to those that met CAPs guidelines (62.61 years), and also observed a significantly larger lesion size (1.95 cm, *p* < 0.0001). Although this cohort is small, the earlier age, the larger size of lesions detected, and the detection of one invasive tumor and several high-grade IPMNs seems relevant when considering expanding current screening guidelines to include patients at an increased risk for disease that are on the cusp of reaching current CAPS guidelines. Moreover, a personalized approach that also examines individual patient characteristics such as hereditary cancer disorders, medical conditions such as pancreatitis, diabetes and obesity, and environmental factors such as smoking, and drinking histories can help to determine whether these people are at a 5% lifetime risk and would benefit from screening.

We also reported on 66 individuals not known to have a familial or hereditary predisposition to PC who presented with incidental findings of pre-malignant pancreatic cysts. This group of “sporadic” HRI had a later age at diagnosis, which may have led to larger lesions than the other two groups, in line with other studies [47,48]. Moreover, this group had the highest percentage (45.5%) of patients requiring surgical resection who had significant pathology. The National Comprehensive Cancer Network (version 1.2018) recently started recommending that genetic counseling and germline testing should be considered for patients with a personal or family history of PC or those with a clinical suspicion of inherited susceptibility. Data from a recent case-control study support this recommendation, as germline mutations in one of six genes (*CDKN2A*, *TP53*, *MLH1*, *BRCA2*, *ATM*, and *BRCA1*) were identified in 5.5% of all PC patients, including 7.9% of patients with a family history of PC and 5.2% of patients without a family history of PC [49]. Taken together, this group with sporadic findings of pre-malignant pancreatic cysts may also represent a population for whom germline genetic testing for cancer predisposition genes should be offered.

Our study is limited because it is based on a retrospective cohort of individuals from a single institution. Despite this, our cohort is unique because it includes patients from two protocols that recruited participants based on their family history and/or presence of a known germline mutation and another protocol that recruited participants unselected for family history or genetic profile. In doing so, we included a diverse cohort of HRI from our comprehensive cancer center. Additionally, it is very possible that a subset of HRI from this cohort has been followed for their medical management or received genetic counseling and testing at other centers and that our information is incomplete. Thus, further follow-up of this cohort is warranted to update their medical and screening history. Moreover, results of this follow-up could inform a prospective study that could serve to verify this retrospective review. Despite these limitations, our study identifies a number of areas in which we can work to optimize guidelines for who is offered genetic counseling and testing and screening. Large multi-center trials are needed to validate our findings and better define the optimal screening regimen for various groups of HRI, with an overarching goal of promoting the detection and treatment of pre-malignant lesions and early, operable disease.

## 4. Methods

Data was gathered from three IRB-approved protocols based at Moffitt Cancer Center: the Inherited Cancer Registry (ICARE) Initiative (MCC 12347, PI: C. Laronga), the High Risk Surveillance Protocol (MCC 14882, PI: J. Klapman) [6], and the Florida Pancreas Collaborative (MCC 18336, PI: J. Permuth) [50]. The ICARE initiative aims to establish a registry of individuals interested in participating in studies of the genetic causes of cancer and seeks to evaluate the roles of genetic and environmental risk factors in the development of tumors or related conditions. Eligible individuals joined ICARE between July 2010 and March 2017 at Moffitt Cancer Center. The High Risk Surveillance Protocol provides annual EUS (with fine needle aspiration if possible) for individuals meeting CAPs guidelines and took place between June 2007 and December 2017 [7]. To be eligible, individuals must have two or more relatives with PDAC with at least one FDR affected, and be at least 40 years old or 10 years younger than the youngest affected family member, have Peutz-Jeghers Syndrome and be at least 30 years old, have hereditary pancreatitis, have familial atypical multiple mole melanoma syndrome, or have a *BRCA2* mutation and at least one FDR or SDR with documented PC [6]. The Florida Pancreas Collaborative is an ongoing biorepository that was established in September of 2015 and includes individuals newly-diagnosed with a breadth of pancreatic conditions ranging from early and late-stage PDAC; benign, pre-malignant, and malignant pancreatic cysts; and pancreatitis [50].

Eligible HRI were identified from the aforementioned protocols and data was collected from the electronic medical record on variables including: age, gender, race/ethnicity, abdominal imaging modalities used and at what age, type of lesion detected, age at which lesion was detected, surgical pathology, and vital status. Data was also collected on personal medical history, body mass index, and history of tobacco exposure and alcohol use. When recording the type of lesion detected, the diagnosis was abstracted from the first imaging report on which the lesion was found or an ambulatory care note. Comparisons were made across different at-risk groups using chi-squared test and Fisher’s exact test (where the cell size was <5) for categorical variables and ANOVA for continuous variables.

## Supplementary Material

Supplementary Materials

## Figures and Tables

**Table 1.** Known hereditary cancer syndromes or syndromes involving chronic inflammation, the relative lifetime risk for PC, and reports of PC precursor lesions among mutation carriers [14].

Syndrome	Gene(s)	Risk of PC by Age 70–75	Studies Reporting PC Precursor Lesions * among Carriers
Hereditary Breast and Ovarian Cancer (HBOC)	*BRCA2*	4.5–8%	[23]
	*BRCA1, PALB2*	3.6%	
Peutz-Jeghers (PJS)	*STK11/LKB1*	36%	[24]
Familial atypical multiple-mole melanoma (FAMMM)	*P16/CDKN2A*	13–17%	[25]
Hereditary non-polyposis colorectal cancer (HNPCC)	*MSH2, MLH1, MSH6, PMS1, PMS2*	3.7%	[23,26,27]
Familial adenomatous polyposis (FAP)	*APC*	1.7%	[28]
Ataxia telangiectasia	*ATM*	<5%	None identified
Li Fraumeni	*TP53*	<5%	[29]
Hereditary Pancreatitis	*PRSS1, SPINK1*	25–54%	[30]
Cystic Fibrosis	*CFTR*	<5%	[31]

* Estimates in this column pertain to intraductal papillary mucinous neoplasms (IPMNs), the cystic PC precursor most commonly detected via imaging.

**Table 2.** Characteristics of the high risk cohort, stratified by group. Note: High risk individuals that met CAPS guidelines had either ≥2 FDR or a known hereditary cancer disorder and a relative with pancreatic cancer. Increased risk and sporadic cases did not meet CAPS guidelines for screening. The increased risk group had either 1 FDR or a germline mutation with no family history of pancreatic cancer. Sporadic cases did not have any family history or known genetic mutation.

Characteristic	Entire Cohort	High Risk According to CAPS Guidelines (*n* = 105)	Increased risk that Does Not Meet CAPS Guidelines (*n* = 158)	Sporadic Cases (*n* = 66)	*p* *
**Sex**					
Male	86 (26.1)	33 (31.4)	21 (13.3)	32 (48.5)	<0.0001
Female	243 (73.9)	72 (68.6)	137 (86.7)	34 (51.5)	
**Race/ethnicity**					
Non-Hispanic White	277 (84.5)	93 (88.5)	127 (80.9)	57 (86.4)	0.86
Non-Hispanic Black	18 (5.5)	5 (4.8)	10 (6.4)	3 (4.5)	
Hispanic White	22 (6.7)	5 (4.8)	13 (8.3)	4 (6.1)	
Other	11 (3.3)	2 (1.9)	7(4.4)	2 (3.0)	
Missing	1	0	0	1	
**Smoking Status**					
Current	27 (8.2)	9 (8.9)	14 (10.2)	4 (6.3)	0.06
Former	192 (58.4)	23 (22.8)	33 (23.9)	27 (42.9)	
Never	83 (25.2)	69 (68.3)	91 (65.9)	32 (50.8)	
Missing	27	4	20	3	
**Personal History of Cancer or Other Conditions**					
Breast Cancer	10 (3.1)	5 (4.8)	2 (1.3)	3 (4.5)	€
Colorectal cancer	3 (0.9)	0 (0.0)	1(0.6)	2 93.0)	
^¥^ Gynecological Cancer	3 (0.9)	1 (1.0)	0 (0.0)	2 (3.0)	
Prostate Cancer	10 (3.1)	2 (1.9)	0 (0.0)	8(12.1)	
^¥^ Other cancer	13 (4.0)	1 (1.0)	2 (1.3)	10 (15.2)	
Pancreatitis	9 (2.8)	2 (1.9)	2 (1.3)	5 (7.6)	
Diabetes	9 (2.8)	1 (1.0)	1 (0.6)	7 (10.6)	
None	270 (82.6)	92 (88.5)	149 (94.9)	29 (43.9)	
Missing	2	1	1	0	
**Pancreatic lesion detected on imaging**					
Had imaging and a lesion was detected	107 (32.5)	31 (29.5)	10 (6.3)	66 (100.0)	<0.0001
Had imaging and no lesion was detected	65 (19.8)	52 (49.5)	13 (8.2)	0 (0.0)	
No imaging on file	157 (47.7)	22 (21.0)	135 (85.4)	0 (0.0)	
**Type of pancreatic lesion detected**					
Cyst, type not specified	49 (14.9)	20 (19.1)	2 (1.3)	27 (40.9)	<0.001^†^
Intraductal papillary mucinous neoplasm	51 (15.5)	9 (8.6)	8 (5.1)	34 (51.5)	
Mucinous cystic neoplasm	1 (0.3)	0 (0.0))	0 (0.0)	1 (1.5)	
Pseudocyst	2 (0.6)	0 (0.0)	0 (0.0)	2 (3.0)	
Solid Tumor/Adenocarcinoma	3 (0.9)	2 (1.9)	0 (0.0)	1 (1.5)	
Pancreatic Neuroendocrine Tumor	1 (0.3)	0 (0.0)	0 (0.0)	1 (1.5)	
**Average age at diagnosis of pancreatic lesion**	65.5	62.6	60.7	67.6	<0.020
**Average size of largest lesion detected (cm)**	2	0.7	2	2.6	<0.0001
**Body Mass Index (BMI)**	-	28.3	30.3	27	0.27

* When calculating *p*-values, missing values were not included. (No percentages were calculated for missing values.);

€ *p* value could not be reliably estimated due to the small sample sizes in each group.

¥ Gynecological cancer includes ovarian cancer, uterine cancer, or cervical cancer. Other cancers included melanoma, bladder cancer, lung cancer, anus cancer, lymphoma, throat cancer, thyroid cancer, and tongue cancer.

† *p* value estimated by collapsing data into three categories: cyst, type not specified; IPMN; and all others.
