# Supplementary material for: Characteristics and Clinical Outcomes of Individuals at High Risk for Pancreatic Cancer: A Descriptive Analysis from a Comprehensive Cancer Center
Source: Gastrointest Disord (Basel). Author manuscript; Available in PMC 2020 Jun 29. (PMC7324042; doi:10.3390/gidisord1010008)
Supplement: Supplementary Materials [file NIHMS1572548-supplement-Supplementary_Materials.pdf]

Supplementary

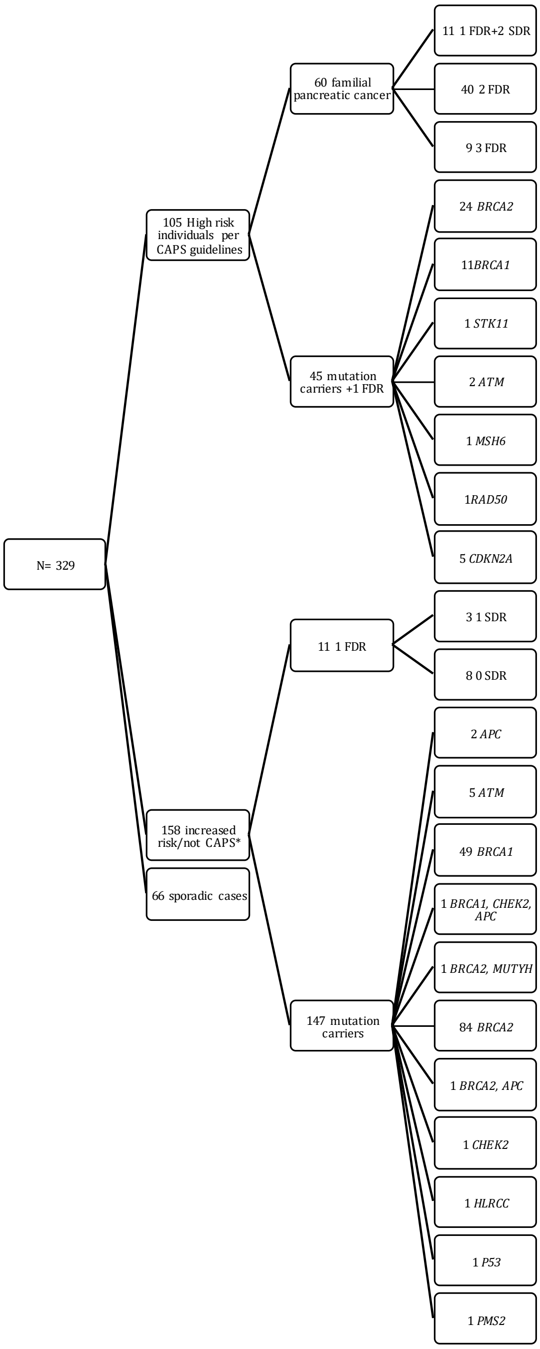

Figure S1. Breakdown of the study cohort. High risk individuals fulfill the CAPS guideline for being at high risk for PDAC, elevated risk individuals have an increased risk for PDAC but do not meet the CAPs guidelines, and sporadic cases have no known deleterious mutation or family history of PDAC.
